# On the dynamics and control of mechanical properties of hierarchical rotating rigid unit auxetics

**DOI:** 10.1038/srep46529

**Published:** 2017-04-26

**Authors:** Krzysztof K. Dudek, Ruben Gatt, Luke Mizzi, Mirosław R. Dudek, Daphne Attard, Kenneth E. Evans, Joseph N. Grima

**Affiliations:** 1 Metamaterials Unit, Faculty of Science, University of Malta, Msida MSD 2080, Malta; 2 Institute of Physics, University of Zielona Gora, ul. Szafrana 4a, 65-069 Zielona Gora, Poland; 3 College of Engineering, Mathematics and Physical Sciences, University of Exeter, Exeter EX4 4QF, UK; 4 Department of Chemistry, Faculty of Science, University of Malta, Msida MSD 2080, Malta

## Abstract

In this work, we investigate the deformation mechanism of auxetic hierarchical rotating square systems through a dynamics approach. We show how their deformation behaviour, hence their mechanical properties and final configuration for a given applied load, can be manipulated solely by altering the resistance to rotational motion of the hinges within the system. This provides enhanced tunability without necessarily changing the geometry of the system, a phenomenon which is not typically observed in other non-hierarchical unimode auxetic systems. This gives this hierarchical system increased versatility and tunability thus making it more amenable to be employed in practical application which may range from smart filtration to smart dressings.

The design of multifunctional materials entails a clear understanding of their underlying physics. Mechanical metamaterials which exhibit a negative Poisson’s ratios (auxetics)^1^,^2^,^3^,^4^,^5^,^6^,^7^,^8^,^9^,^10^,^11^,^12^,^13^ have recently been the focus of many studies due to the various enhanced properties they exhibit, the relative ease with which they may be produced, and the potential applications where they may be used. For example, it has been shown that auxeticity can be achieved through the introduction of cuts and perforations^14^,^15^,^16^, molding^17^ and 3D printing^18^ techniques which are well established within the field of engineering. This also permits the production of custom-designed systems even at industrial scales at a relative low cost. Of particular interest are auxetics incorporating elements of hierarchy^19^,^20^,^21^,^22^, with the hierarchical rotating square structure proposed by Cho *et al*.^23^ and Gatt *et al*.^24^ being one of the most prominent examples of these systems. It is composed of rotating ‘square units’ which are made up of an array of smaller rotating units themselves, a process which in theory may be repeated indefinitely. The inclusion of hierarchy in the classic rotating square system^25^ has been shown to result in increased versatility, expandability and enhanced tunability of phononic wave propagation^23^,^24^,^26^,^27^.

Despite all this, no attempts have yet been made to study the dynamic behaviour of the mechanical properties of these hierarchical systems, meaning that some of the more fundamental properties have hitherto not been explored. For example, the current methods of analysis give no information on the rate of deformation meaning that it is impossible to estimate the time required for a system to move from one conformation to another, or the path the system takes as a function of time during this deformation.

In view of the above, the deformation behaviour of a hierarchical structure based on the rigid rotating units model (see Fig. 1) was investigated through a set of dynamics equations which take into consideration the rotational motion of the units in the respective hierarchical levels. Each structure considered in this work consists of individual but identical squares connected together by means of identical hinges at their vertices that offer the same resistance to rotation. Such hinges can be controlled through friction (e.g. pin-jointed structures) or more complex hinges such as those described through a harmoic potential (giving a more realistic description of a material). Using this dynamics approach, the mechanical versatility of these hierarchical structures was studied for systems having the same initial geometry but with each system having hinges that offer different resistance to rotational motion (when compared to other systems) albeit each hinge having the same properties within the same system. Such control of the resistance to rotational motion could be achieved through the use of ‘intelligent hinges’. Such ‘intelligent hinges’ could be designed in a way so that the resistance to rotational motion is controlled through the application of electromagnetic fields and/or temperature changes. For example, if the hinges are produced from materials having different co-efficient of thermal expansion, a small change in temperature would result in different stiffness of the hinge. This means that even after fabrication, the stiffness of the hinges could be changed by changing the magnitude of the temperature. An analogical approach could involve the insertion of electromagnets on the rotating units in which case the neighbouring units would repel or attract each other which effectively would alter the resultant stiffness of the hinges. A similar concept concerning the use of magnets to deform a mechanical system was proposed by Grima *et al*.^2^

All this contrasts with previous works on these systems^23^,^24^,^26^,^27^ which have focused mainly on the geometric versatility and implementation of these frameworks as perforated systems. This results in hierarchical systems with increased tunability which may be employed in numerous applications, including smart dressing and smart filters.

## Model

**Figure 1:**
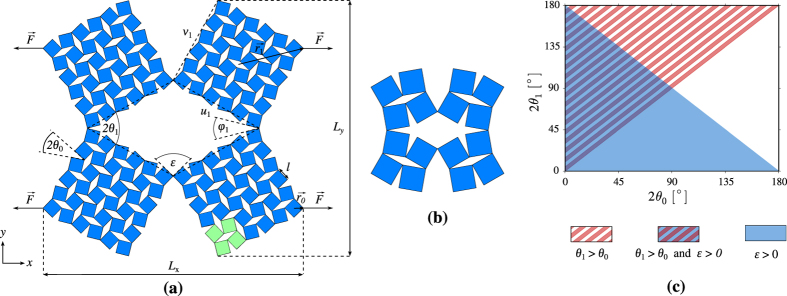
The panels in this figure present (**a**) the two-level hierarchical auxetic system with four square-like units corresponding to Level 1 of the structure, where each unit consists of *N*_0_ × *N*_0_ (in the provided example *N*_0_ = 3) Level 0 repeat units (bright green), (**b**) an example of the structure corresponding to *N*_0_ = 1 and (**c**) the permissible angles for *θ*_0_ and *θ*_1_, which conditions ensure that the squares do not overlap whith each other and the system retains the same connectivity. This is attained when conditions *θ*_1_ > *θ*_0_ and *ε* = *π* − 2*θ*_1_ − 2*θ*_0_ > 0 are satisfied.

In this paper, a model specifically designed to describe the dynamic behaviour and predict the mechanical properties of the hierarchical system shown in Fig. 1(b) will be presented and discussed. This may be described as a finite two-level hierarchical system having four square-like units in the upper level (corresponding to Level 1 of the structure) each of which is made from four other sqaures (the Level 0) having a linear dimension of *l*. This sytem is a particular case of a more general two-level heirarchical system where each of the Level 1 building blocks consists of 2*N*_*o,x*_ × 2*N*_*o,y*_ squares, where *N*_*o,x*_ and *N*_*o,y*_ stand for the number of Level 0 units in the two orthogonal directions associated with a Level 1 building block, see Fig. 1(a). Note that if *N*_*o,x*_ = *N*_*o,y*_ = *N*_0_, the Level 0 would approximate the shape of a square (as is the case in the present work) whilst if *N*_*o,x*_ ≠ *N*_*o,y*_, then the Level 0 would assume the shape of a rectangle.

Variables *θ*_0_ and *θ*_1_ correspond to angles between the adjacent units of the zeroth and first level respectively. These quantities can in turn be used in order to determine the linear dimensions (*L*_*x*_ and *L*_*y*_) of the discussed system (see Supplementary Information). It is also very important to note that the hierarchical systems considered in this study cannot be constructed for any combination of *θ*_0_ and *θ*_1_. The possible values of these angles are given in Fig. 1(c).

This model operates under the assumption that the Level 0 rotating square units of the system are completely rigid and cannot distort or change shape in any way during deformation. The validity or otherwise of this assumption for a real physical system will depend on how the real system is constructed. For example, this model derived here is likely to be always valid for systems constructed from stiff units (e.g. metal) connected through hinges, i.e. a system which is purposely constructed to satisfy these assumptions of the model. For small strains, the ‘rigid units’ assumption is also expected to be applicable to other systems, such as perforated systems as described in Cho *et al*.^23^ and Grima *et al*.^28^, where previous studies have shown that the smaller the ‘connection’ between the squares in relation to the size of the units (e.g. squares) themselves, the more valid the assumption is^28^. Furthermore, the Level 0 squares and Level 1 building blocks will be assumed to be connected together through hinges which permit relative rotation of two connected units. The symmetry of the system, as well as its geometric constraints result in a system which only has few degrees of freedom. In particular, under the condition of uniaxial on-axis loading, all of the units constituting the *i*-th level of the system are geometrically constrained to rotate by the same angle, while the 0-th level squares remain rigid, i.e. they do not distort or change shape in any way. Under these conditions, for a given value of *l* (size of the length of a single square), the geometry of the whole system can be described through just two independent variables, angles *θ*_0_ and *θ*_1_. This means that it is sufficient to investigate the rotation of individual units in both levels in order to obtain a complete picture of the deformation mechanism of the considered structure, i.e. the dynamics of the system may be fully described through a set of equations which are the rotational analogues of Newton’s equation of motion, which equations may be solved numerically. In other words, the deformation through time, of the whole system may be established by solving the equations governing the changes in the angles *θ*_*i*_ at a given time, an approach which allows for perfect rigidity of the 0-th level rotating units and is highly efficient from a computational time point of view, as the number of equations which must be solved at a given time corresponds to the number of levels within the system and is nearly independent of the number of units constituting a given level. This stems from the fact that the number of rigid units corresponding to the *i*-th level is only a number parameter in the equation describing the dynamics of the angle *θ*_*i*_, as will be discussed later in this paper.

In order to induce a deformation in the systems discussed in this work, a force 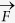 is applied on each of the leftmost and rightmost vertices of the system, as shown in Fig. 1(a) (or topmost and bottommost, depending on the direction of loading).

Resistance of the hinge to rotation may be defined in a number of ways depending on the way how the hinges within the considered system are constructed. A very well-known approach makes use of a harmonic potential in order to describe the energetics governing the hinging process, an approach which can mimic a number of realistic scenarios, including the behaviour at small strains of perforated and other similar systems corresponding to an analogical geometry.

In the considered model based on a harmonic potential, it is assumed that resistance to rotation is solely due to a restoring force associated with hinges. In other words, apart from assuming that Newton’s equations are adequate to describe the dynamics of the discussed system, it is also being assumed that the system is just being uniaxially stretched and not being subjected to additional factors (magnetic field, medium viscosity etc.). The approach used here is expected to be valid for a wide range of macroscale systems present in a medium with relatively low viscosity which are not subjected to additional environmental factors. At this point it is important to note that, in the case of this particular potential, even if all of the hinges within the system are the same, the restoring force corresponding to hinges from Level 0 and Level 1 may vary as angles *θ*_0_ and *θ*_1_ might be opened to a different extent with respect to equilibrium angles 2*θ*_0,*eq*_ and 2*θ*_1,*eq*_ respectively. In such a case, the resistance to rotation associated with individaul hinges may be quantified in terms of torques 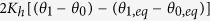 or 
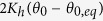
 depending on the position within the system. In this case *K*_*h*_ stands for a stiffness constant associated with the resistance torque. Furthermore, based on Fig. 1(b), one may note that for the considered structure there are always four hinges corresponding to Level 1 of the system and by extension to the torque 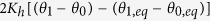. Under such assumptions, for a given level to keep expanding, the resultant resistance torque associated with this level has to be overcome by the torque corresponding to the force applied to the system at all times. The deformation of the system depends on the collective magnitude of torques associated with the Level 0 squares (*S*_0_) and the Level 1 subunits (*S*_1_), as follows:


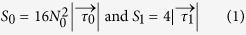


where, 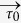 stands for the torque associated with each of the Level 0 squares and analogically, 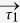 corresponds to the torque associated with the Level 1 building block. In this case, the value of *S*_*i*_ depends on the angular acceleration 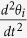 (which is induced by the force 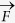) and the moment of inertia *I*_*i*_ corresponding to the *i*-th level, hence it can be defined as 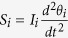. The deformation of the system can then be established through the rotation of the units found in each level, by means of the rotational analog of Newton’s equation of motion:


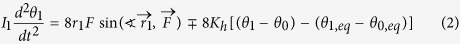



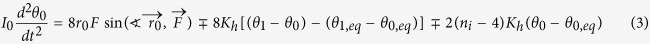


where, *n*_*i*_, is the number of hinges corresponding to Level *i* of the system, *r*_*i*_, is the distance between the vertex where the force is applied and the centre of mass of the Level *i* building block.

In this study, a two-level system is being considered and therefore *i* = 0, 1. Here, *I*_0_ corresponds to the rotation of individual squares with respect to their own centres and *I*_1_ is associated with the rotation of the Level 1 building blocks with respect to their centres of mass. The information concerning the way how these quantities were calculated can be found in the Methods section.

The signs in eqs 2 and 3, are set in a way so that the resultant resistance torque always opposes the rotational motion of units corresponding to the *i*-th level. Furthermore, the factor 8 in eqs 2 and 3 is associated with the way the force is being applied to the system and the resulting reaction forces as described in ref. 29.

It is important to note that the methodology developed here can be applied to various other analogous constructs including other hierarchical systems composed of an arbitrary type of rotating rigid units. Furthermore, it is important to highlight the fact that although the model proposed above is based on a specific type of hinge in which the rotational motion is governed by a harmonic potential, the derived model can be used for an arbitrary potential or type of interaction. A very good example can be hinges where the rotational motion is governed by friction. In this case, one can assume that for such a model, the resistance to rotation is solely due to friction from all the hinges in the system, which are identical to each other irrespective of their position within the system. In this case, the resistance to rotation may be quantified in terms of a friction torque *f* = *f*_*i*_ (*i* = 0,1) resulting from the friction caused by the rotational motion of the hinge, where the value of *f* remains constant regardless of changes in angular velocity and angle of aperture of the subunit. Under such assumptions, for a given level to start deforming, the resultant friction torque associated with this level has to be overcome by the torque corresponding to the force applied to the system. In this case, the Newton’s equation of motion describing rotational motion of the *i*-th level, can be written in an analogical manner as was the case for the harmonic potential as follows:


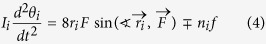


Systems having hinges governed by friction, apart from being indisputably simple to describe mathematically, are a very good representation of macroscopic pin-jointed structures where the resistance to rotation of the hinge is associated with the friction governing the hinging process.

## Results and Discussion

**Figure 2:**
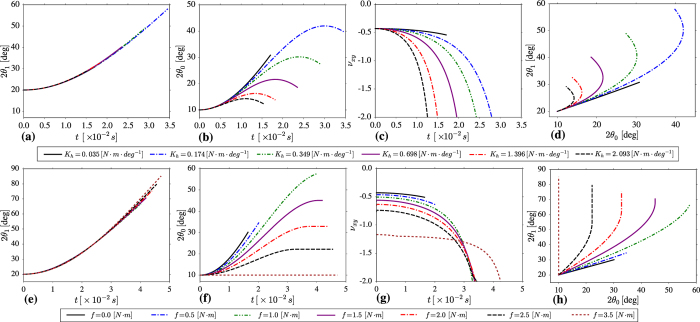
Plots showing the variation in (**a**) *θ*_1_, (b) *θ*_0_ and (**c**) Poisson’s ratio *v*_*xy*_ as a function of time *t* for loading in the *x* direction for systems with *K*_*h*_ values ranging from 0.035 N · m · deg^−1^ to 2.093 N · m · deg^−1^ and (**d**) the relation of *θ*_1_ to *θ*_0_ for a deforming structure having the motion of the hinges governed by harmonic potential. Similarly, plots (**e**,**f** and **g**) show the variation in *θ*_1_, *θ*_0_ and *v*_*xy*_ respectively as a function of time in the case of friction-based hinges corresponding to the value of *f* ranging between 0 N · m and 3.5 N · m. (**h**) Shows the relation of *θ*_1_ to *θ*_0_ for a deforming structure having the motion of the hinges governed by friction. In all cases considered, *N*_0_ = 1 and *F* = 500 N. It is important to note that in the case of (**c** and **g**), the scale in the y-axis (incremental Poisson’s ratio) was arbitrarily stopped at −2, since this value of the Poisson’s ratio tends to −∞ upon approaching the maximum deformation. A cut-off value of −2 is appropriate in view of the fact that the part of the deformation which is not included in panels (**c** and **g**) is relatively small, as shown in Table 2. An analogical set of results, plotted with respect to applied strain, is provided in the Supplementary Information.

Numerical solutions of the model presented above suggest that irrespective of the hinge being used, a tensile force results in the system deforming through a relative rotation of the constituent units where, for a given initial structure, the actual manner of deformation, and hence the Poisson’s ratio, is dependent on the resistance to motion offered by the hinges. This means that if the magnitude of the resistance to rotational motion offered by the hinges could be controlled, then it would be possible to control the mechanical behaviour of the system without altering its geometry. Such control over the resistance to the rotational motion of the hinges can be achieved through the use of magnetic fields (which concept was first proposed by Grima *et al*.^30^) or thermal expansion of hinges. This is very significant as it is the first time that a change in the Poisson’s ratio is not being imparted through a change in the geometry of the system, as is normally the case, but merely due to a change in the resistance associated with the hinges of the hierarchical system. This is clearly shown by the results plotted in Fig. 2 for systems with the same initial geometry, set to *l* = 0.05 m, *N*_0_ = 1, *θ*_0_ = 10° and *θ*_0_ = 20°, having hinges which offer different resistance to motion. Here it is important to note that the changes in the mechanical properties of the hierarchical systems occur while the system is still deforming via the rotating mechanism. This is completely different from the effect observed in other auxetic systems such as hexagonal re-entrant honeycombs^31^,^32^,^33^, where the mechanical properties depend on the interplay between the three main deformation mechanisms present in these systems, i.e. the stretching, hinging and flexing mechanisms. For such re-entrant systems one could alter the mechanical properties by changing the ratio of their respective stiffness constants. However in our case, there is no such interplay of mechanisms and the change in mechanical properties was obtained solely as a result of the relative rotations of Level 0 and Level 1 units.

**Figure 3:**
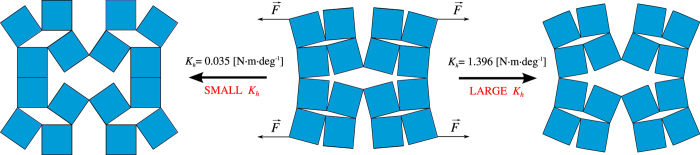
Diagrams showing the final state of the deformation of two systems, where the hinging process is governed by friction, with *K*_*h*_ values of 0.035 N · m · deg^−1^ and 1.396 N · m · deg^−1^. Animations presenting the entire deformation range of these systems are provided in the Supplementary Information: ANIM1.gif and ANIM2.gif respectively.

Figure 2(a–d) show the results for systems where the resistance to motion is governed through a harmonic potential associated with every hinge within the system. These different structures have the same initial geometry but different values of a stiffness constant *K*_*h*_ ranging from 0.035 N · m · deg^−1^ to 2.093 N · m · deg^−1^. In all cases, the system was subjected to a constant force having a magnitude of 500 N until it was geometrically locked i.e. until the system could not continue to deform through rotations of the Level 1 and/or Level 0 quadrilaterals. A detailed analysis of these results indicates that in the case of the hierarchical systems having relatively high values of *K*_*h*_, Level 1 opens to a greater extent than Level 0 of the structure. This can be concluded from the fact that angle *θ*_1_ opens to a greater extent than angle *θ*_0_ as shown in Fig. 2 and Table 1. This is in accordance with the numerical and experimental work conducted by Gatt *et al*.^24^ and Tang *et al*.^26^ concerning the deformation of hierarchical perforated materials. However this deformation behaviour is not observed for all systems with the equivalent initial geometric configuration. In fact, for systems having relatively low values of *K*_*h*_, the opposite behaviour is observed, with the Level 0 squares opening to a greater extent than the larger Level 1 units. Therefore, these results suggest that the deformation behaviour depends on the magnitude of the *K*_*h*_ coefficient (or other parameter depending on the type of hinge). This is very important as it indicates that for a given initial conformation, the final loaded structure depends upon the value of the hinge resistance to the rotational motion coefficient, as indicated in Fig. 3. It is also important to note that although *θ*_1_ always increases, for relatively low values of *K*_*h*_, referring to Figs 1(a) and 3, angle *ϕ*_1_ becomes smaller with time whilst for relatively large values of *K*_*h*_ this angle becomes increasingly larger. It is also important to note that the deformation behaviour (and hence mechanical properties) of this hierarchical system can also be controlled through the magnitude of the force applied. In particular, for a system having a constant resistance to rotational motion, an increase in the magnitude of the force results in Level 0 quadrilaterals opening to a greater extent in comparison to Level 1 quadrilaterals, without changing the structure itself i.e. without having to deconstruct and re-construct the structure with different hinges. Additional fine-tuning may also be achieved by a applying a force which changes in magnitude with time (see Supplementary Information).

This difference in the deformation mechanisms upon altering the *K*_*h*_ coefficient may be explained if one considers the number of hinges present within each level of the system. The deformation of the rotating units in the respective levels is governed by the ratio of the resultant friction torque in the zeroth and first level of the system. For the hierarchical structure considered above, the total number of hinges present in Level 0 (*n*_0_) is equal to 20 whilst the total number of hinges present in Level 1 (*n*_1_), is equal to 4. Based on eqs 2 and 3, the resultant resistance torque, which the system has to overcome in order to expand, depends on the number of hinges present within each level of the system. Hence, in the considered case of *N*_0_ = 1, the corresponding resistance torque of the zeroth level of the structure is significantly larger than in the case of the first level, which stems from the fact that the number of hinges corresponding to Level 0 is five times greater than it is the case for Level 1. Thus for relatively large values of *K*_*h*_, for example *K*_*h*_ = 1.396 N · m · deg^−1^, the magnitude of the resultant resistance torque corresponding to Level 0 is relatively large when compared to that of the torque associated with the applied force. At the same time, Level 1 units are not as affected by the value of *K*_*h*_ (see Fig. 2(a)) due to the fact that in this case, the resistance torque associated with only 4 hinges has to be overcome. This results in a greater deformation of the Level 1 units. However, in cases where the *K*_*h*_ constant assumes a relatively small value (such as in the case of *K*_*h*_ = 0.035 N · m · deg^−1^), the resultant resistance torque becomes insignificant in comparison to the one associated with an applied force and the distance *r*_*i*_ becomes the governing factor for the deformation of the system. This means that the Level 0 units deform to a greater extent in the case of a relatively small value of *K*_*h*_. The effect which the value of *K*_*h*_ has on the deformation of the system is clearly shown in Figs 2(d) and 3 where the final configuration of the system corresponding to both of the discussed values of *K*_*h*_ is presented. Based on Fig. 2(b), one can note that Level 0 starts closing during the process of deformation. This result is associated with the fact that the resultant restoring force (for this level) is greater than in the case of Level 1 (number of hinges is greater for Level 0 than it is the case for Level 1), hence it is more prone to exceed the torque corresponding to the external force. In order to better understand the extent of deformation for each of the considered paramaters, the analogical set of results plotted with respect to strain is provided in the Supplementary Information.

The above results indicate that one may control the deformation behaviour of the system simply by changing the magnitude of the coefficient corresponding to the resistance to the rotational motion (such as *K*_*h*_ in the case of a harmonic potential). This also suggests that for any two-level hierarchical rotating squares geometry (assuming that both levels open at the same time), there is a specific value of *K*_*h*_ where the rate of angle opening of *θ*_0_ and *θ*_1_ is equal. Such a treshold value of *K*_*h*_ can be denoted as *K*_*h,T*_. It can be obtained by means of eqs 2 and 3, where by assuming that *ω*_1_ = *ω*_0_, the value of *K*_*h,T*_ can be found once the condition 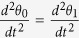 is satisfied. It is also important to note that the value *K*_*h,T*_, can be determined only for a given time since it varies as the angles change.

Figure 2(c), shows the change in the incremental Poisson’s ratio with time for the hierarchical structure under consideration having different values of *K*_*h*_. The incremental Poisson’s ratio^34^, also known as the Poisson’s function^35^ was used in this study as it gives a much better indication of changes in the lateral dimension of the system as a measure of applied stress (i.e. during the deformation) when compared to the engineering Poisson’s ratio. From Fig. 2(c), it is clear that the value of the Poisson’s ratio at a particular time, depends on the value of *K*_*h*_. This stems from the fact that the geometry of Level 1 units (defined by *u*_1_ and *ν*_1_ in Fig. 1(a)) can be described as rectangles rather than as squares, with *u*_1_ = 01083 m and *v*_1_ = 0.0996 m. For relatively large values of *K*_*h*_, *θ*_0_ changes to a small extent, meaning that the dimensions of the Level 1 units remain roughly constant throughout the deformation of the hierarchical structure. It is well known that the Poisson’s ratios of the rotating rectangles model is dependent on the dimensions of the rectangles and the angle between them, where the incremental Poisson’s ratio exhibit an asymptote-like behaviour (*v*_*xy*_ → ±∞) upon approaching the locking conformation, i.e. upon reaching the maximum applied strain in the loading direction. For relatively small values of *K*_*h*_, (for example *K*_*h*_ = 0.035 [N · m · deg^−1^]) *θ*_0_ changes to a large extent, meaning that the dimensions of the Level 1 units change throughout the deformation of the hierarchical structure. This means that in this case, the Poisson’s ratio of the hierarchical system will depend on the relative changes of *θ*_0_ and *θ*_1_. Furthermore, one may note that the initial value of the Poisson’s ratio is the same for all the systems considered (governed by a harmonic potential) irrespective of the value of the stiffness constant. This effect can be explained by the fact that in the case of the harmonic potential, at the time equal to zero, the term corresponding to the restoring torque (see Eqs 2 and 3) assumes a value of zero as the hinges are in their equilibrium states.

**Figure 4:**
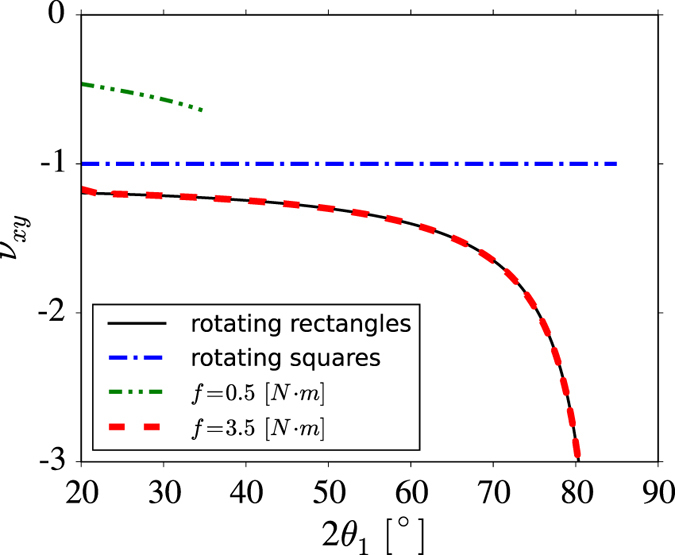
A plot showing a comparison of the Poisson’s ratios obtained from the numerical solutions presented here for *f* = 0.5 N · m and *f* = 3.5 N · m with those calculated from analytical models for uni-level rotating rigid rectangle and square systems.

At this point, it is important to note that the results presented above relate only to a hinge governed by the harmonic potential, but the findings that the deformation pathway and mechnaical properties are affected by the properties of the hinges is a general result. As an example, a similar set of results was produced for structures in which the hinging process is governed by a friction rather than a harmonic potential. The values of *f* (associated with the friction of a hinge) for these structures were set in the range between 0 N · m and 3.5 N · m, while the same geometric parameters considered for the hinges governed by harmonic potential were used. From the results obtained, see Fig. 2(e)–(h), one can note that even though the deformation patterns are slightly different than it was the case for Fig. 2(a)–(d) (which stems from a different nature of the hinging process), both of the systems lead to the same conclusions as analogical trends can be observed in both sets of figures. For example, for relatively high values of *f*, Level 0 of the hierarchical system opens to a smaller extent than Level 1 and, vice-versa, for relatively low values of *f*, Level 0 opens to a greater extent [Table t2]than Level 1 (see Table 3). Furthermore, in the case of the friction-based hinges, the initial Poisson’s ratio is not the same for different values of *f*, which was not the case for the hinges governed by harmonic potential (see Fig. 2(c,g)). This is due to the fact that the initial system varies for different values of *f* as a different resistance to motion has to be overcome from the beginning of the deformation. In fact, referring to Fig. 4, it can be shown that for the hierarchical system having *f* = 3.5 N · m, the Poisson’s ratio follows the same profile as the rigid rotating rectangles model proposed by Grima *et al*.^36^. This result also proves the suitability of this dynamics method to model systems based on rigid rotating units.

Also, although the results discussed here are specific to a particular geometry, the same trends in deformation and Poisson’s ratios are expected to occur for other initial geometric conformations of the hierarchical system, as shown in the Supplementary Information. Furthermore, this result is expected to be valid for larger values of *N*_0_. However, one may presume that as *N*_0_ increases, Level 0 becomes increasingly rigid meaning that the Poisson’s ratio would increasingly depend on the deformation of the Level 1 units. Moreover, as *N*_0_ → ∞, the geometry of Level 1 units resembles more closely that of a square and thus the Poisson’s ratio of the system would approach −1 as expected for the rotating squares model^25^.

All this is very significant, since the work presented here shows that it is possible to alter the deformation mechanism, and hence the mechanical properties of a hierarchical rotating rigid unit system simply by changing the resistance to rotational motion of all the hinges in an even manner. This property is not observed in currently known non-hierarchical rotating rigid unit systems and adds another element of versatility to this class of auxetic structures, which has already been shown through previous studies to possess the potential to exhibit a considerable range of mechanical properties through geometric variation alone^23^,^24^,^26^. This means that if, for example, one were to build a hierarchical system where the Level 0 squares are connected together through ‘smart/intelligent’ hinges with tuneable friction coefficients, one could achieve a considerable range of negative Poisson’s ratio without altering the initial geometry of the system. Moreover, the examples discussed here only provide a glimpse of the true potential of these systems. A greater degree of versatility is envisaged if other geometries besides the rotating square motif are employed and if the number of hierarchical levels in the system is increased. This increased versatility could make these systems ideal for a number of niche applications such as smart filters, where the friction coefficients of ‘intelligent’ hinges may be customized according to the required pore sizes. This way, one filter may be used to filter a range of substances with different parameters, hence reducing material costs. Also, such a filter would be much easier to clean than a normal filter due to the adjustable pore size. Such systems with tunable porosity could also find applications in the design and manufacture of smart dressings as discussed elsewhere^24^,^37^.

## Conclusions

In conclusion, through a dynamics approach, a model was designed to predict the Poisson’s ratio and quantify the relative rotations of the units at each hierarchical level for a hierarchical rotating rigid unit systems. It was shown that unlike unilevel systems, the Poisson’s ratios (including the auxetic potential) and final geometrical configuration for a given applied load of such hierarchical structures may be altered solely by changing the relative resistance to the rotational motion of the hinges of the systems. This contrasts sharply with the behaviour of other auxetic systems where, unless the geometry of the system is altered, changes in the mechanical properties can only be attained through changes in the interplay of different deformation mechanisms. This is very significant as it suggests that if one were to construct such a system through the use of hinges with tuneable friction torques, one may be able to control with ease the relative deformations of the various hierarchical levels and hence the overall macroscopic and mechanical properties of the system. It is hoped that this work will lead to further interest in the field of hierarchical auxetic systems and, in the future, even lead to the production of ‘smart’ hierarchical auxetic systems with tuneable deformation behaviour.

## Methods

### Calculation of the moment of inertia corresponding to Level 0 and Level 1 of the system

The moment of inertia *I*_0_ associated with Level 0 of the structure can be considered as the sum of moments of inertia of all of the Level 0 squares rotating with respect to their centres. Assuming that the rotating rigid units are made of a material having a surface density *ρ*, the moment of inertia of a single square can be expressed as 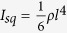. In such a case, *I*_0_ can be calculated by means of the following expression: 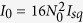.

The moment of inertia *I*_1_, can be defined as four times the moment of inertia corresponding to the Level 1 building block (*I*_1,BB_) rotating with respect to its centre of mass (there are 4 Level 1 building blocks). The moment of inertia *I*_1,BB_ depends on the contribution from each of the Level 0 squares, constituting the Level 1 building block and the distance from the centre of mass of the Level 1 building block to the centre of the particular square within the considered Level 1 unit *d*_*m*_. Thus, *I*_1_ can be expressed as follows:


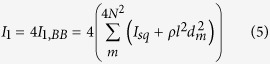


### Numerical solutions

In order to investigate the deformation behaviour of the discussed system, the Newton’s equation of motion proposed in Eq. 4 was written in the form of the following equations:


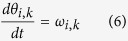



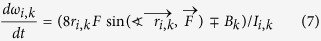


where, *i* = 0, 1 and *k* is the number of a given time step. The *B*_*k*_ variable stands for the magnitude of the resultant torque corresponding to the resistance to rotational motion of hinges. Equations 6 and 7 were solved numerically by means of the classical fourth-order Runge-Kutta numerical algorithm^38^, which is one of the most common methods of solving ordinary differential equations. The initial conditions concerning the value of *θ*_*i*,0_ and *ω*_*I*_,_0_ are provided in the Parameters subsection.

At each time step *k* the following conditions have to be satisifed:


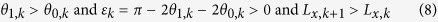


### Calculation of the Poisson’s ratio

In general, for loading in the *x* direction, the Poisson’s ratio can be expressed as follows:


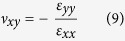


where *ε*_*xx*_ and *ε*_*yy*_ are the strains in the *x* and *y* directions respectively. In this work, the value of *ν*_*xy*_ at a given time was calculated using the following formula:


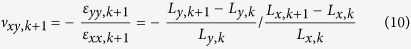


### Parameters

In order to investigate the deformation behaviour of the discussed system for loading in the *x*-direction, a structure consisting of 1 × 1(*N*_0_ × *N*_0_) Level 0 building blocks was used. The constants characterising this system were set as follows: *F* = 500 N, *l* = 0.05 m, *ρ* = 3000 kg · m^−2^ (density of material making up the Level 0 subunits), *ω*_1_ and *ω*_0_ = deg · s^−1^ (the initial angular velocity of the rotating units in the respective first and zeroth levels), Δ*t* = 10^−7^ s. In addition, the initial geometric parameters of the system were set as: 2*θ*_1_ = 20°, 2*θ*_0_ = 10°, which in case of *N*_0_ = 1 leads to, *L*_*x*_ = 0.248 m and *L*_*y*_ = 0.234 m. Furthermore, in order to show how the deformation of the system changes upon varying the value of *K*_*h*_, *K*_*h*_ was set to be equal to {0.035, 0.174, 0.349, 0.698, 1.396, 2.093}N · m · deg^−1^.

In the case of the structure constructed by means of hinges governed by friction, all of the parameters, with the exception of the *f* variable, were kept fixed. In the considered cases *f* assumed the values of {0.0, 0.5, 1.0, 1.5, 2.0, 2.5, 3.5}N · m.

## Additional Information

**How to cite this article:** Dudek, K. K. *et al*. On the dynamics and control of mechanical properties of hierarchical rotating rigid unit auxetics. *Sci. Rep.* **7**, 46529; doi: 10.1038/srep46529 (2017).

**Publisher's note:** Springer Nature remains neutral with regard to jurisdictional claims in published maps and institutional affiliations.

## Supplementary Material

Supplementary Information

Supplementary Video 1

Supplementary Video 2

## Figures and Tables

**Table 1:** Difference between the final and initial value of the angle *θ*_*i*_ for particular values of *K*_*h*_.

*K*_*h*_ [J deg^−1^]	0.035	0.174	0.349	0.698	1.396	2.093
2*θ*_0,*final*_ − 2*θ*_0,*initial*_ [deg]	20.811	29.766	17.233	8.600	3.780	2.231
2*θ*_1,*final*_ − 2*θ*_1,*initial*_ [deg]	10.812	38.101	29.261	20.175	12.661	9.302

In all of the considered cases, the system was subjected to a constant force having a magnitude *F* = 500N throughout the whole process of deformation, i.e. from 2*θ*_*i,initial*_ up to 2*θ*_*i,final*_, when the system goes to the locked conformation.

**Table 2 t2:** Values of the strain (*ε*_*x*_) corresponding to the minimum value of the Poisson’s ratio presented in Fig. 2 in comparison to the final value of the strain (*ε*_*x,final*_) associated with the geometric lockage of the system.

*f* [Nm]	*ν*_*xy*_	*ε*_*x,final*_	*K*_*h*_[N · m · deg^−1^]	*ν*_*xy*_	*ε*_*x,final*_
0.0	−0.508 at *ε*_*x*_ = 0.157	0.157	0.035	−0.543 at *ε*_*x*_ = 0.162	0.162
0.5	−0.640 at *ε*_*x*_ = 0.194	0.194	0.174	−2.0 at *ε*_*x*_ = 0.253	0.271
1.0	−2.0 at *ε*_*x*_ = 0.289	0.303	0.349	−2.0 at *ε*_*x*_ = 0.199	0.212
1.5	−2.0 at *ε*_*x*_ = 0.273	0.291	0.698	−2.0 at *ε*_*x*_ = 0.134	0.143
2.0	−2.0 at *ε*_*x*_ = 0.246	0.266	1.396	−2.0 at *ε*_*x*_ = 0.181	0.086
2.5	−2.0 at *ε*_*x*_ = 0.211	0.234	2.093	−2.0 at *ε*_*x*_ = 0.057	0.062
3.5	−2.0 at *ε*_*x*_ = 0.183	0.187	—	—	—

**Table 3:** Difference between the final and initial value of the angle *θ*_*i*_ for particular values of *f*.

*f* [N·m]	0.0	0.5	1.0	1.5	2.0	2.5	3.5
2*θ*_0,*final*_ − 2*θ*_0,*initial*_ [deg]	20.133	24.686	47.658	35.075	22.869	12.176	0.034
2*θ*_1,*final*_ − 2*θ*_1,*initial*_ [deg]	10.134	14.688	46.793	50.492	55.351	59.799	65.182

In all of the considered cases, the system was subjected to a constant force having a magnitude *F* = 500 N throughout the whole process of deformation, i.e. from 2*θ*_*i,initial*_ up to 2*θ*_*i,final*_, when the system goes to the locked conformation.
