# Supplementary material for: On the dynamics and control of mechanical properties of hierarchical rotating rigid unit auxetics
Source: Sci Rep. 2017 Apr 26;7:46529. doi: 10.1038/srep46529 (PMC5405418; doi:10.1038/srep46529)
Supplement: Supplementary Information [file srep46529-s1.pdf]

Supplementary Information

On the dynamics and control of mechanical properties of hierarchical rotating rigid unit auxetics

K.K. Dudek[1,2], R. Gatt*[1], L. Mizzi[1], M.R. Dudek[2], D. Attard[1], K.E. Evans[3], and J.N. Grima[1,4]

[1]Metamaterials Unit, Faculty of Science, University of Malta, Msida MSD 2080, Malta
[2]Institute of Physics, University of Zielona Góra, ul. Szafrana 4a, 65-069 Zielona Góra, Poland
[3]College of Engineering, Mathematics and Physical Sciences, University of Exeter, Exeter EX4 4QF, UK
[4]Department of Chemistry, Faculty of Science, University of Malta, Msida MSD 2080, Malta

1 Expressions for L_x and L_y

The geometry of a two-level hierarchical rotating square system may be described by the aperture between the Level 0 squares ($2\theta_0$), the aperture between the Level 1 square-like units ($2\theta_1$) and the number of Level 0 repeat units (N_0). This means that the Level 1 building block is made up of $N_0 \times N_0$ Level 0 repeat units which corresponds to $2N_0 \times 2N_0$ squares. These parameters may be used to define the linear dimensions of the Level 1 square-like units, u_1 and v_1, as follows: $u_1 = 2N_0 l \cos(\theta_0) + 2(N_0 - 1) l \sin(\theta_0)$ and $v_1 = 2N_0 l (\cos(\theta_0) + \sin(\theta_0))$, where l stands for the length of the sides of the Level 0 squares. These dimensions can in turn be used to define the overall on-axis dimensions of the system:

$$L_x = 2\left(u_1 \cos(\theta_1) + v_1 \sin(\theta_1)\right) \tag{1}$$

$$L_y = 2\left(v_1 \cos(\theta_1) + u_1 \sin(\theta_1)\right) \tag{2}$$

2 Dependence of values of θ_0 and θ_1 on strain ε_x

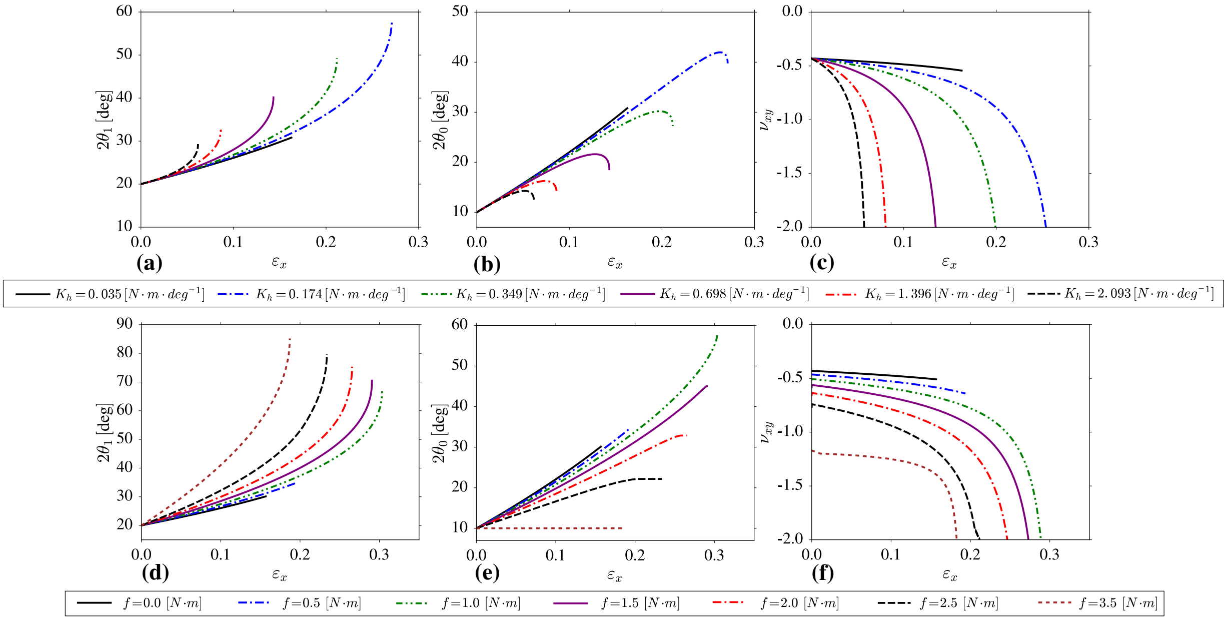

FIG. S 1: Behaviour of the system plotted with respect to the strain (ε_x). Panels (a)-(c) correspond to the results where the resistance to the rotational motion of hinges was governed by the harmonic potential while panels (d)-(f) correspond to friction-based hinges. All of these results were generated for the analogical parameters as it was the case in the main text.

Fig. S 1 provides an additional information regarding the magnitude of strain corresponding to each of the cases considered in the main text.

3 The effect of the variation of the magnitude of the force F in time on the behaviour of the system

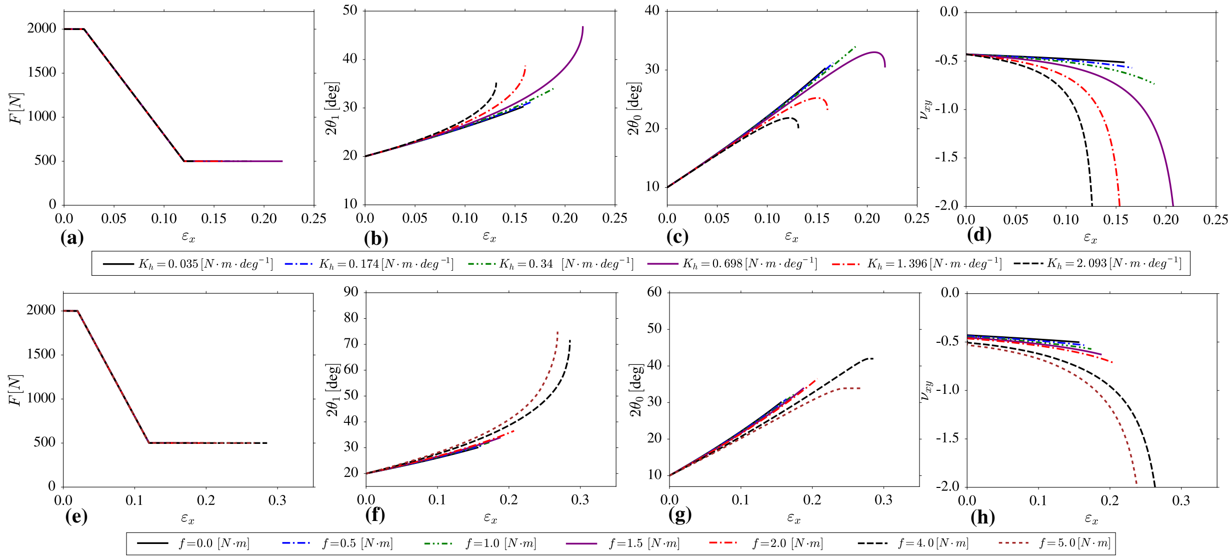

FIG. S 2: Deformation of the system (expressed in terms of the change in strain) subjected to the force F having its magnitude gradually changed from 2000N to 500N (which value was considered in the main text). Panels (a)-(d) correspond to the system with hinges having their rotational motion governed by the harmonic potential while panels (e)-(h) correspond to friction-based hinges.

The effect of a non-constant force on the deformation of the hierarchical system under study was also investigated. This was done by changing the magnitude of the force $\overrightarrow{F}$ as shown in Fig. S 2(a), (e) and Fig. S 3(a), (e). These results indicate that although there are similar trends to the results obtained for the system subjected to the constant force $\overrightarrow{F} = 500N$), in this case, Level 0 opens to a greater extent for the same coefficients corresponding to the resistance to the rotational motion (See Fig. S 2). This is due to the fact that in this case the initial force is very high and thus, the ratio of the torque generated by the applied force and the resistance torque is significantly greater than it was the case for a constant force $\overrightarrow{F} = 500N$. This also indicates that one may apply a force which changes in magnitude with time in order to have an additional way of controlling the behaviour of the system.

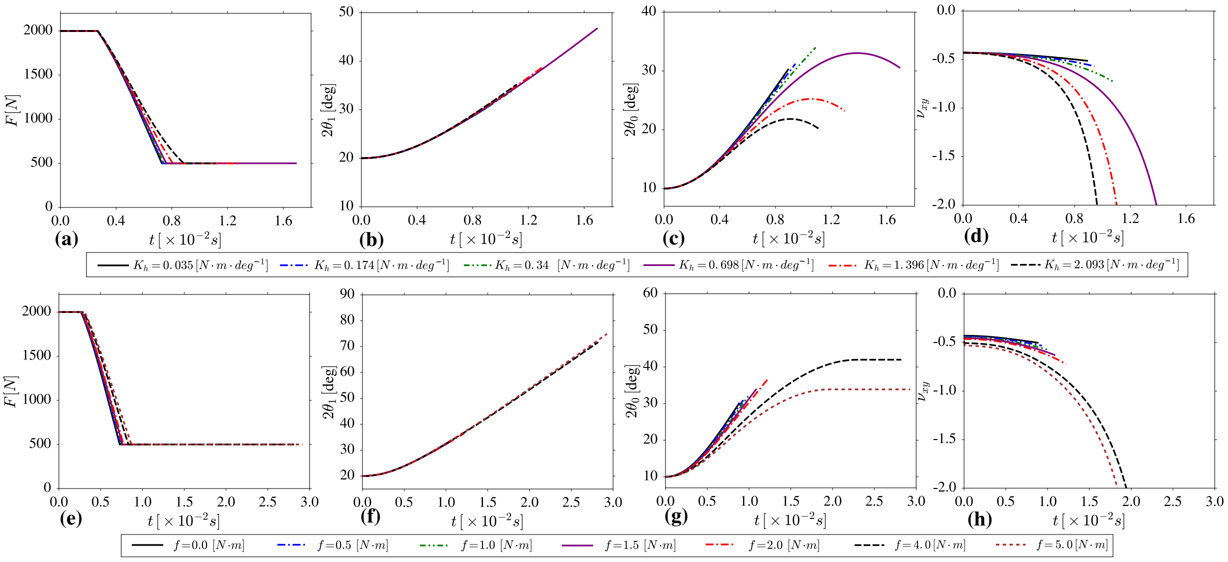

FIG. S 3: Deformation of the system (expressed in terms of the change in time) subjected to the force F having its magnitude gradually changed from 2000N to 500N (which value was considered in the main text). Panels (a)-(d) correspond to the system with hinges having their rotational motion governed by the harmonic potential while panels (e)-(h) correspond to friction-based hinges.

4 Dependence of strain on time for all of the considered types of hinges

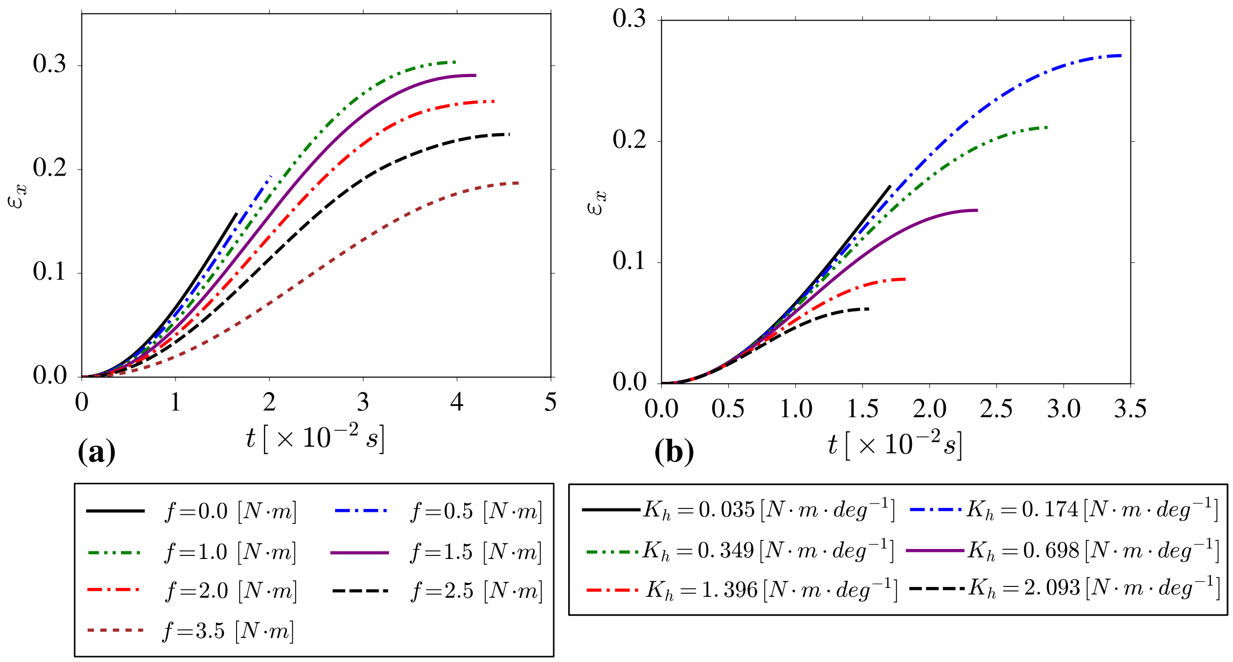

FIG. S 4: Dependence of the value of the strain on time for the considered types of hinges. Results shown in panel (a) correspond to the hinges in which the resistance to the rotational motion is governed by friction whereas the results in panel (b) are associated with the harmonic potential.

5 Results for different initial configurations of θ_0 and θ_1 taking the friction-based hinge as an example

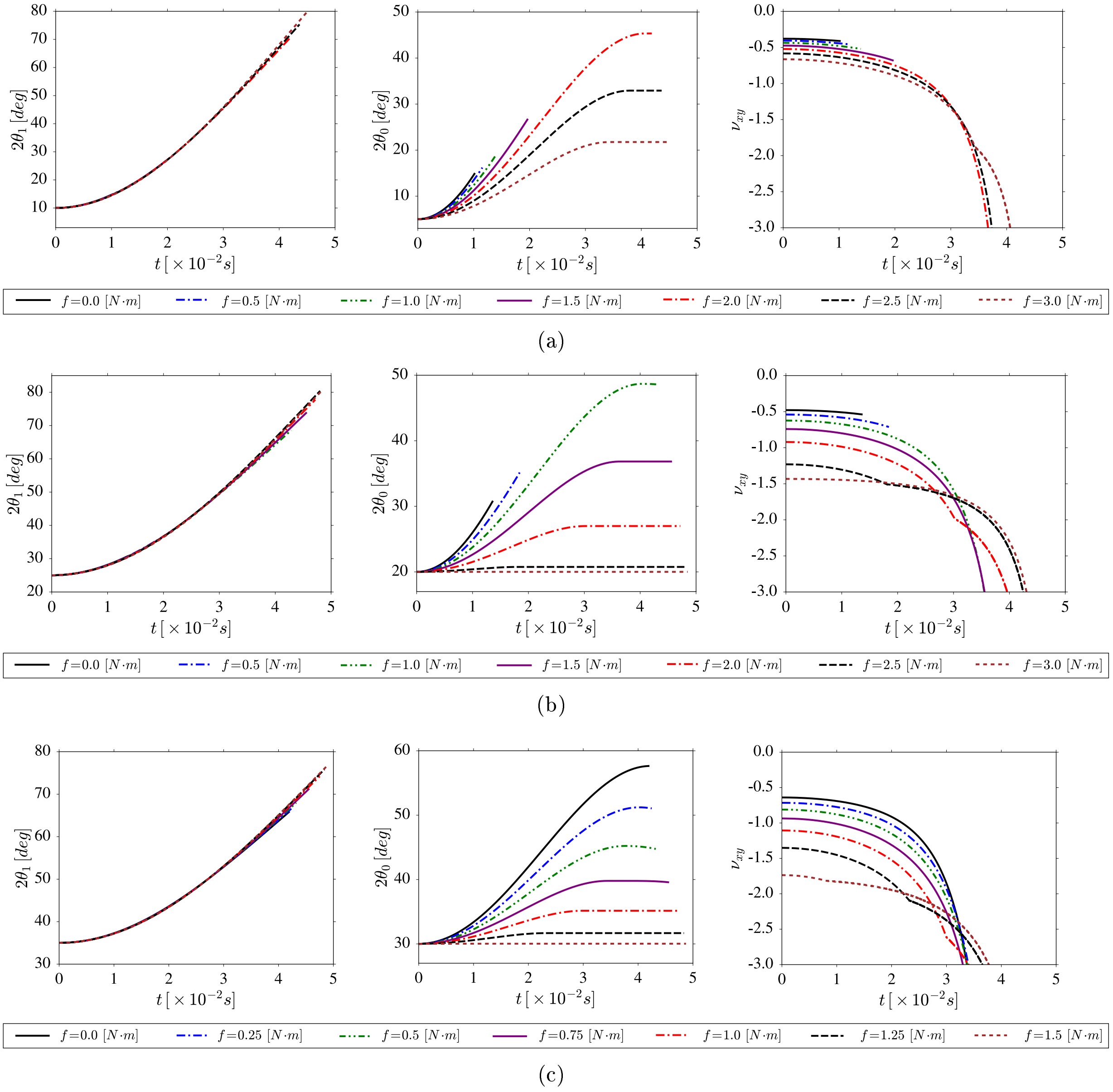

FIG. S 5: Behaviour of the structure in the case of different initial configurations associated with values of angles θ_0 and θ_1. The panels in this figure correspond to (a) $2\theta_0 = 5°$, $2\theta_1 = 10°$, (b) $2\theta_0 = 20°$, $2\theta_1 = 25°$ and (c) $2\theta_0 = 30°$, $2\theta_1 = 35°$. All of the results presented here are associated with $N_0 = 1$. Moreover, the results were generated for hinges governed by friction.

Based on Fig. S 5(a), one can note that irrespective of the initial values of angles θ_0 and θ_1, the pathway of θ_1 with respect to t is closely resembling the one presented in the main text. The same applies to Fig. S 5(b) and Fig. S 5(c), where analogically to the results in the main text, which shows that upon increasing the value of f the rate of deformation of the angle θ_0 decreases and that the Poisson's ratio becomes increasingly more negative. All of this leads to the conclusion that the results discussed in the main text are not dependent on the initial value of the θ_0 and θ_1 parameters.

6 Different loading directions

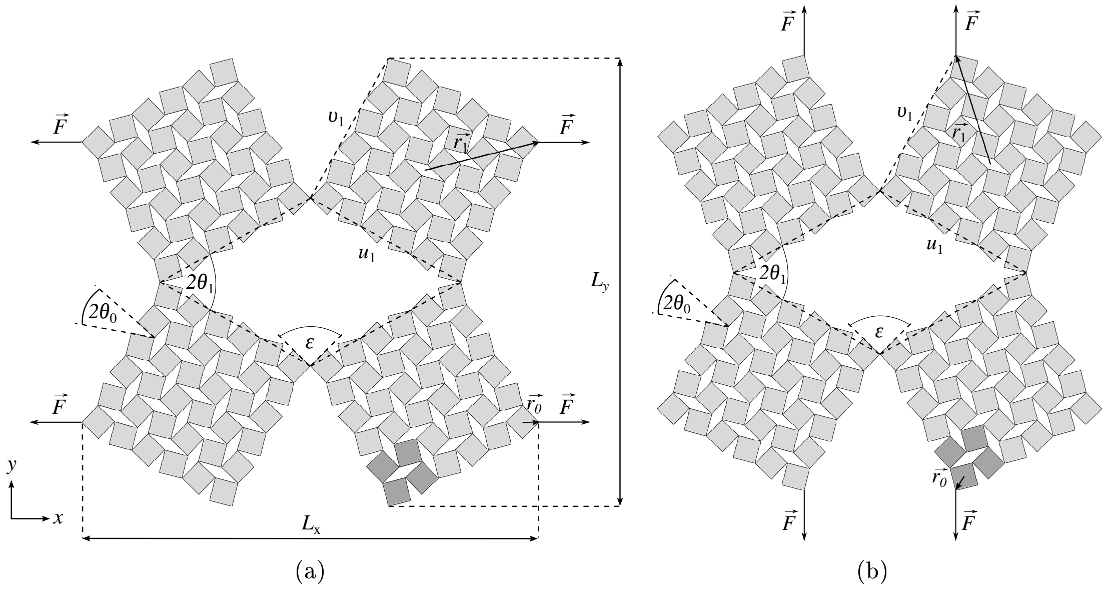

FIG. S 6: The system considered in this study for loading (a) in the x-direction and (b) in the y-direction.

In the main paper only loading in the x-direction (see Fig. S 6(a)) was discussed. This is because for this type of connectivity, upon subjecting the structure to the uniaxial tensile load, both levels can open simultaneously. On the other hand, for loading in the y-direction, the Level 0 squares tend to close when subjected to the same tensile load, while at the same time, Level 1 building blocks rotate in a way promoting the expansion of the structure in the loading direction. This effect can be explained based on the orientation of vectors $\overrightarrow{r_i}$ and $\overrightarrow{F}$.

Based on Fig. S6(a), one can note that in the case of the vast majority of permissible values of θ_0 and θ_1, the torque corresponding to Level 1 building block and the Level 0 squares to which the force is applied, has the same orientation. Based on the right hand rule, it is easy to note that in both cases a clockwise rotation will occur. This in turn leads to opening of both levels upon the application of the tensile load. In the case of connectivity II, Fig. S6(b), in the majority of cases, the right hand rule implies that the Level 0 and Level 1 building blocks will close and open respectively.

7 Results concerning loading in the y-direction taking friction-based hinge as an example

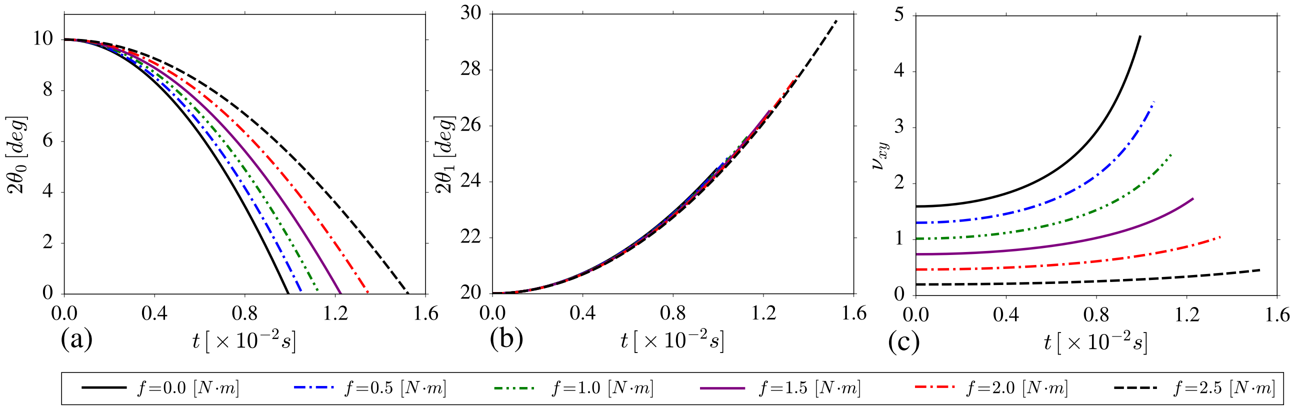

FIG. S 7: Plots showing a variation in (a) θ_0, (b) θ_1 and (c) Poisson's ratio ν_{xy} as a function of time in the loading direction for systems corresponding to type II connectivity with f values ranging between 0.5 N·m and 2.5 N·m. Graphs shown here were generated for initial angles $2\theta_0$ and $2\theta_1$ set to be equal to 10° and 20° respectively.

In the case of loading in the y-direction, whenever angle $\left(\sphericalangle\overrightarrow{r_0}, \overrightarrow{F}\right)$ assumes positive value, the resultant torque associated with Level 0 is directed in a way which promotes a decrease of the θ_0 angle (see Fig. S 7(a)). This is opposite to what can be observed for loading in the x-direction where for positive values of angles $\left(\sphericalangle\overrightarrow{r_0}, \overrightarrow{F}\right)$ and $\left(\sphericalangle\overrightarrow{r_1}, \overrightarrow{F}\right)$ both levels of the system are opening.

8 Animations

ANIM1.gif: Animation showing the deformation of the system corresponding to $K_h = 0.035\,\mathrm{N{\cdot}m{\cdot}deg^{-1}}$, where the hinging process is governed by the harmonic potential.

ANIM2.gif: Animation showing the deformation of the system corresponding to $K_h = 1.396\,\mathrm{N{\cdot}m{\cdot}deg^{-1}}$, where the hinging process is governed by the harmonic potential.
