# Supplementary figures and images for: On the dynamics and control of mechanical properties of hierarchical rotating rigid unit auxetics
Source: Sci Rep. 2017 Apr 26;7:46529. doi: 10.1038/srep46529 (PMC5405418; doi:10.1038/srep46529)

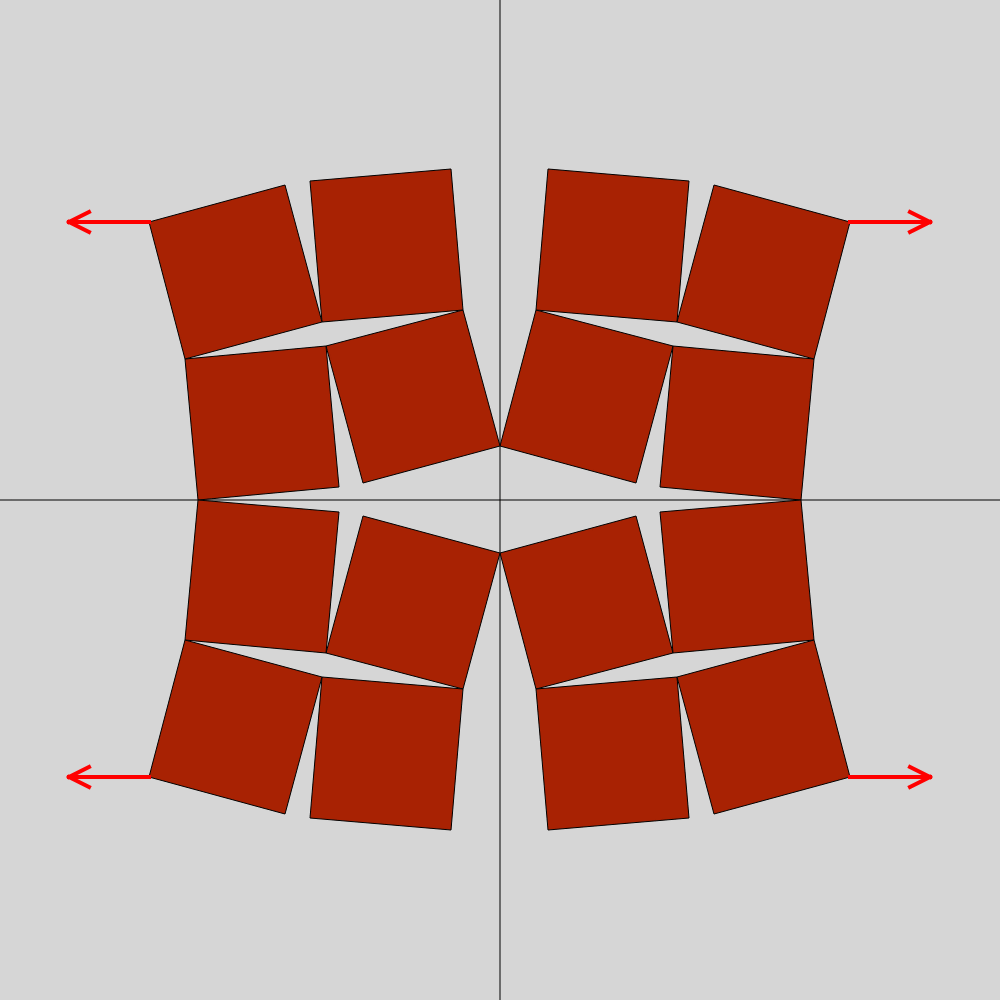

Supplement: Supplementary Video 1 [file srep46529-s2.gif]

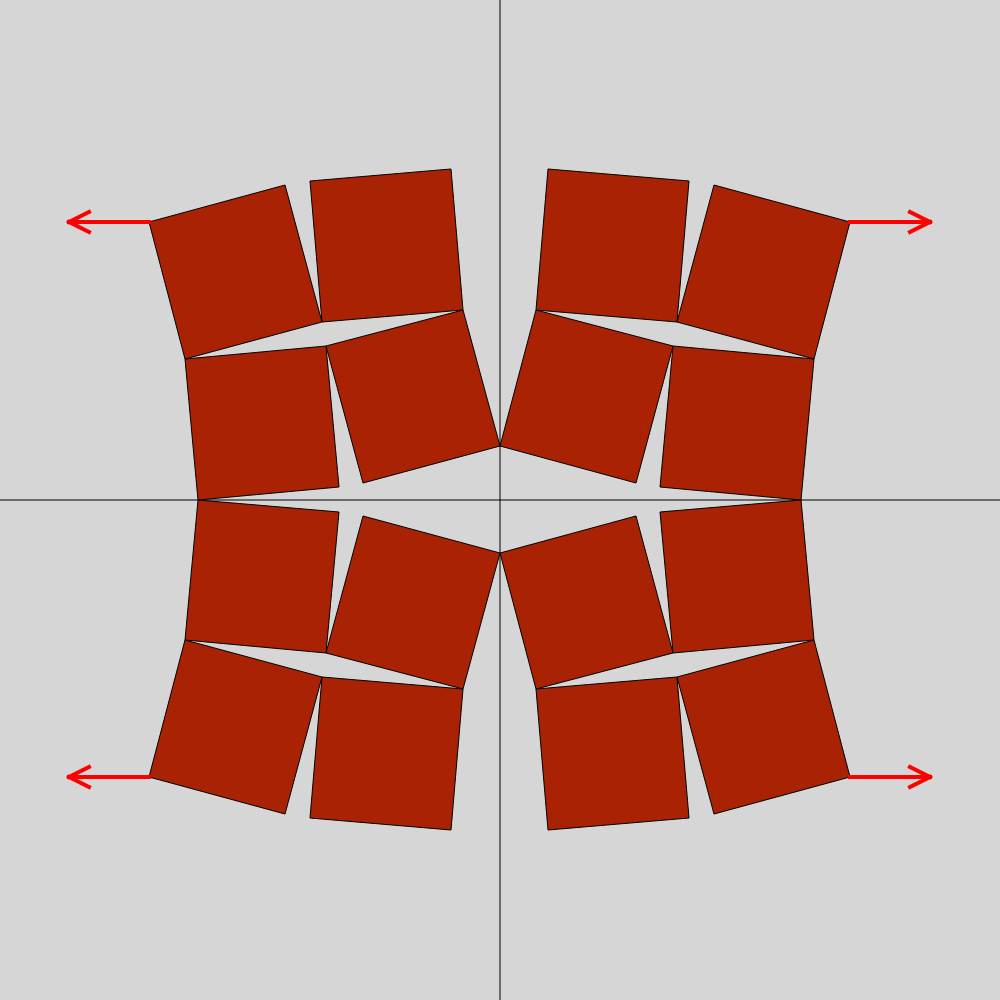

Supplement: Supplementary Video 2 [file srep46529-s3.gif]
